# *Alisma canaliculatum* Extract Affects AGS Gastric Cancer Cells by Inducing Apoptosis

**DOI:** 10.7150/ijms.55212

**Published:** 2021-03-19

**Authors:** Min Ji Kwon, Jeong Nam Kim, Joon Park, Yun Tai Kim, Min Jae Lee, Byung Joo Kim

**Affiliations:** 1 Division of Longevity and Biofunctional Medicine, Pusan National University School of Korean Medicine, Yangsan 50612, Republic of Korea.; 2 Division of Food Functionality, Korea Food Research Institute, Wanju-gun 55365, Republic of Korea.; 3 Department of Food Biotechnology, Korea University of Science & Technology, Daejeon 34113, Republic of Korea.; 4 College of Veterinary Medicine, Kangwon National University, Chuncheon 24341, Republic of Korea.

**Keywords:** *Alisma canaliculatum*, Apoptosis, AGS, Gastric cancer.

## Abstract

The anti-cancer effects of *Alisma canaliculatum* extracts (ACE) were identified in AGS gastric cancer cells. Our results showed that ACE inhibited the growth of AGS cells, increased the proportion of sub-G1 phase cells, and depolarized the membrane potential of mitochondria. ACE-induced gastric cancer cell death was associated with Bcl-2, survivin and Bax level changes, and it activated caspase-3 and -9. In addition, it was involved in the activation of MAPKs and increased the reactive oxygen species (ROS). These results suggest that ACE induces apoptosis in AGS gastric cancer cells, and therefore, ACE may have the potential to treat gastric cancer.

## Introduction

Gastric cancer places a heavy burden on people worldwide and is becoming the most common cancer globally 1, 2. Cancer is almost impossible to cure if not detected early, and as the disease progresses, the pain and economic losses of patients and their families become enormous, resulting in immense social distress 3. Therefore, the importance of cancer prevention and its early treatment has increased recently, since the quality of life, which involves living a pleasant and healthy life, has gained significance over merely extending the life span of patients 4.

*Alisma canaliculatum* is a commonly used traditional medicine in China, Japan, and Korea 5. It exhibits various pharmacological properties, such as anti-bacterial and anti-cancer effects, and protects the liver cells 6-8. It is also effective in treating postmenopausal osteoporosis 9, 10 and suppressing breast cancer metastasis 11.

Apoptosis plays a vital role in maintaining the balance between cell death and cell division. Apoptosis induced abnormalities can result in uncontrolled cell division, leading to diseases such as cancer 12. Thus, apoptosis has become a key tool in cancer treatment, and the mechanism underlying cancer cell growth has become an important target for cancer cell therapy 13. However, the anti-cancer roles of *A. canaliculatum* on gastric cancer cells have not yet been studied. Thus, we investigated the mechanisms underlying the anti-cancer effects of *A. canaliculatum* extract (ACE) on AGS gastric cancer cells.

## Material and Methods

### Materials

*A. canaliculatum* extract (ACE) was purchased from Korea Plant Extract Bank (Cheongju, Korea). Alisol B monoacetate was obtained from Chemfaces (Wuhan, China). High-performance liquid chromatography (HPLC) grade acetonitrile (ACN) and distilled water were purchased from Thermo Fisher Scientific (Santa Clara, CA, USA).

### HPLC analysis

A stock solution of ACE and alisol B monoacetate was prepared by separately dissolving each of them in methanol. Standard calibration solutions were prepared, ranging from 6.25 to 100 μg/mL. The HPLC analysis of the extract was performed using a JASCO HPLC system equipped with a binary pump, an auto-sampler, a column oven, and a UV detector. An aliquot containing 20 μL of standard or ACE sample solutions was directly injected onto a Symmetry300^TM^ C18 column (4.6 × 250 mm × 5 μm). The mobile phase consisted of a mixture of (A) 0.1% phosphoric acid in distilled water and (B) ACN. The gradient running condition was as follows: 40-40% A for 0-10 min, 40-30% A for 10-20 min, 30-20% A for 20-30 min, 20-40% A for 30-35 min, with an assay determination run time of 35 min.

### 3-[4,5-dimethylthiazol-2-yl]-2,5-diphenyltetrazolium bromide (MTT) assay

AGS cells were propagated in RPMI-1640 medium (Gibco-BRL, St. Louis, MO, USA) supplemented with 10% heat-inactivated fetal bovine serum (Invitrogen, Grand Island, NY, USA) containing 1% penicillin/streptomycin (Invitrogen, Grand Island, NY, USA) at 37°C and seeded onto 12-well plates at a density of 3 × 10^5^ cells/well. Cell viability was determined using MTT assay.

### Measurement of cell cycle

AGS cells were treated with ethyl alcohol and vortexed. Samples were centrifuged for 5 min and the supernatant was discarded. Cell pellets were resuspended in propidium iodide (PI) staining solution containing RNase and incubated for 40 min in the dark at room temperature. Samples were analyzed using a fluorescence-activated cell sorter (FACScan; Becton-Dickinson, Mountain View, CA, USA).

### Measurement of mitochondrial depolarization assay

AGS cells were treated with 50 nM tetramethylrhodamine methyl ester (TMRM; Sigma-Aldrich, St. Louis, MO, USA) for 30 min. Fluorescence intensities were measured using a BD FACSCANTO II (BD Biosciences, Sunnyvale, CA, USA) at the excitation and emission wavelengths of 510 and 580 nm, respectively.

### Western blot analysis

The amount of protein extracted from cells was measured using Bradford method. An equal amount of protein (20 μg) from the samples was separated by 8% or 10% SDS-PAGE and probed with specific antibodies. Antibodies against survivin (#2808), ERK (#9102), pERK (#9106), JNK (#9252), pJNK (#9251), p38 (#9212), and pp38 (#9216) were purchased from Cell Signaling Technology (Danvers, MA, USA), and antibodies against Bcl-2 (#sc-783), Bax (#sc-493), caspase-3 (#sc-7148), caspase-9 (#sc-7885), PARP (#sc-7150), β-actin (#sc-47778), and GAPDH (#sc-32233) were procured from Santa Cruz Biotechnology (Santa Cruz, CA, USA).

### Caspase assay

Caspase-3 and -9 assay kits (Cellular Activity Assay Kit Plus; BioMol, Plymouth, PA, USA) were used. After resuspending the cells in ice-cold cell lysis buffer, the supernatant was removed. Supernatant samples were incubated with caspase substrate (400-lM Ac-DEVD-pNA; 50 μl) at 37°C and then, samples were read at 405 nm.

### Measurement of reactive oxygen species (ROS) levels

ROS levels were determined with 2',7'-dichlorodihydrofluorescein diacetate (DCF-DA; Molecular Probes, Eugene, OR, USA). Fluorescence was measured using FACS (Becton-Dickinson, Mountain View, CA, USA), at excitation/emission wavelengths of 488/525 nm.

### Statistics

One-way ANOVA with Tukey's *post hoc* comparison was used for multiple comparisons. The analysis was performed using Prism 6.0 (GraphPad Software Inc., La Jolla, CA, USA) and Origin 8.0 (OriginLab Corporation, Northampton, MA, USA) software. Data were expressed as the mean ± standard error of the mean (SEM), and *P* values < 0.05 were considered statistically significant.

## Results

### Quantification of alisol B monoacetate in ACE by HPLC-UV

**Figure 1:**
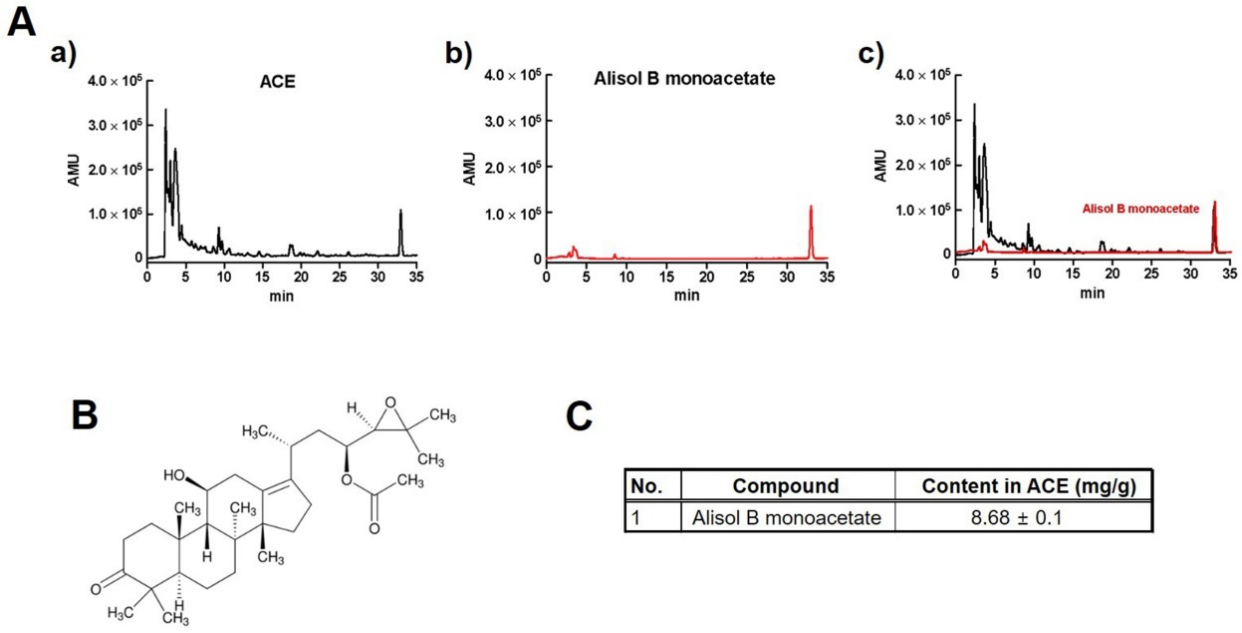
Quantification of alisol B monoacetate in ACE by HPLC-UV. HPLC-UV chromatograms of alisol B monoacetate (A) in 100 mg/L standard solution (middle) and ACE (top). Structural formula (B) and content (C) of alisol B monoacetate in ACE.

A previous study reported that alisol B monoacetate is a major component in ACE 14,15. To quantify alisol B monoacetate in ACE, HPLC-UV was performed (Fig. 1A and 1B). The results showed that alisol B monoacetate was present at a concentration of 8.68 ± 0.1 mg/g in ACE (Fig. 1C).

### Effects of ACE on AGS gastric cancer cells

**Figure 2:**
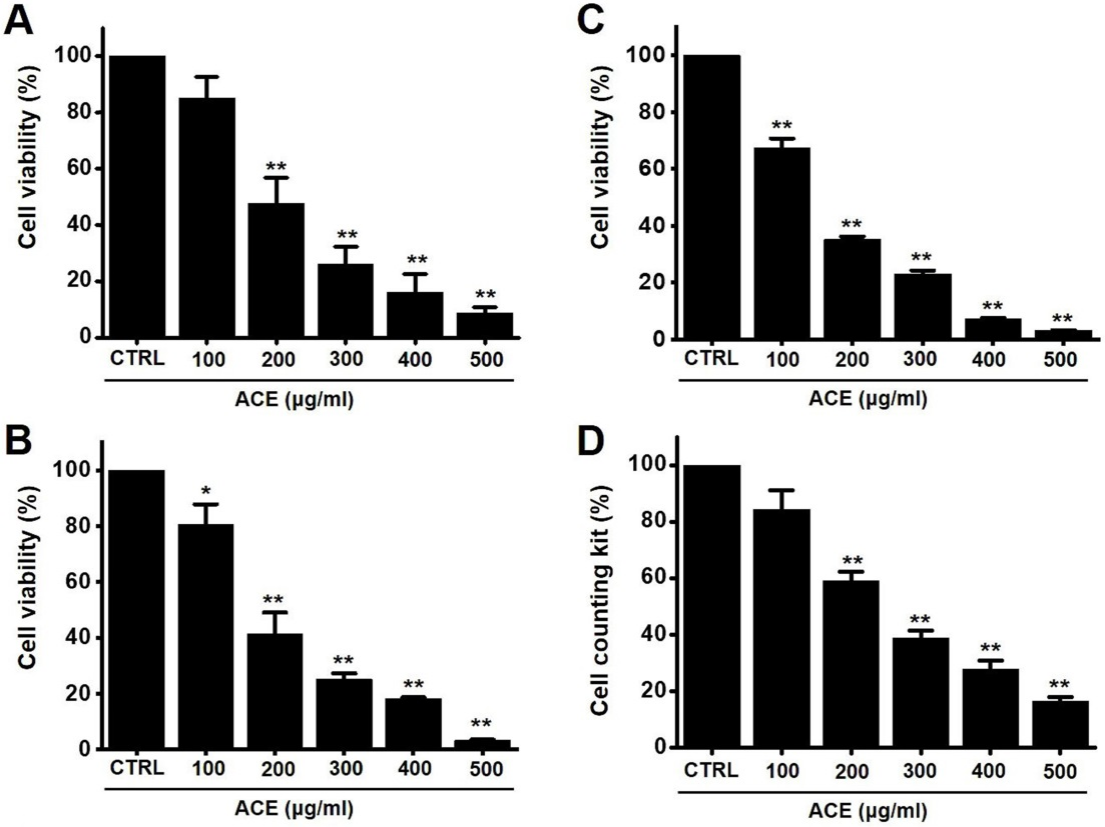
ACE reduces AGS gastric cancer cell viability after 24 h, 48 h and 72 h. Results of (A) 24 h MTT, (B) 48 h and (C) 72 h and (B) CCK-8 (cell counting kit-8) assays showing that ACE reduces AGS cell viability dose-dependently. Results are presented as the mean ± SEM. *p < 0.05. **p < 0.01. ACE, *Alisma canaliculatum* extract; CTRL, control.

**Figure 3:**
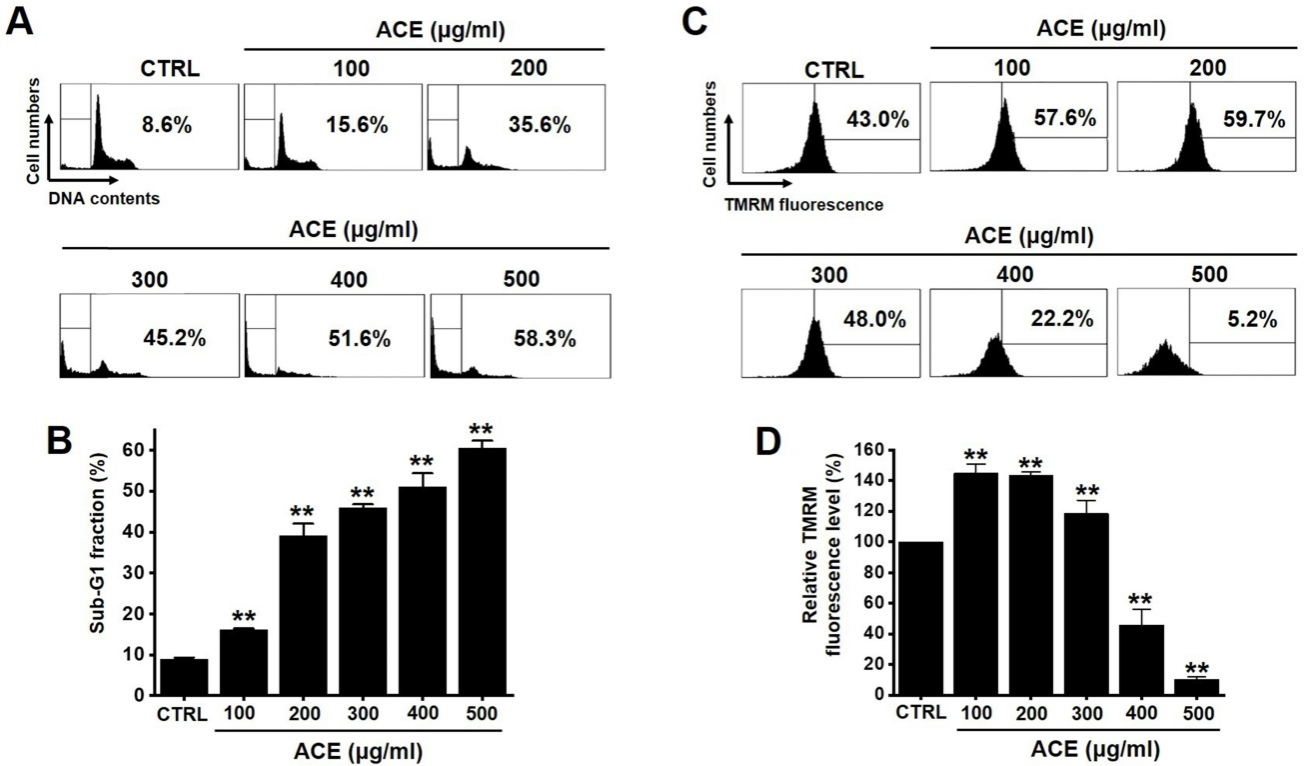
ACE induces an increase in the proportion of cells in the sub‑G1 phase as well as the depolarization of the mitochondrial membrane. (A) Flow cytometric analysis results of the cell cycle and (B) sub-G1 fractions are expressed. (C) Fluorescence of mitochondrial membrane depolarization measured using FACS analysis. (D) Relative mitochondrial TMRM fluorescence levels. Results are presented as the mean ± SEM. **p < 0.01. ACE, *Alisma canaliculatum* extract; CTRL, control.

The MTT method was employed to determine the effects of ACE-induced apoptosis in AGS gastric cancer cells for 24 h. The survival of AGS cells was reduced by 85.1 ± 7.7 % at 100 µg/ml, 47.5 ± 9.3 % (*p*< 0.01) at 200 µg/ml, 26.1 ± 6.4 % (*p*< 0.01) at 300 µg/ml, 16.1 ± 6.6 % (*p*< 0.01) at 400 µg/ml, and 8.8 ± 2.0 % (*p*< 0.01) at 500 µg/ml of ACE at 24 h (Fig. 2A), by 80.5 ± 7.4 % (*p*< 0.05) at 100 µg/ml, 41.5 ± 7.6 % (*p*< 0.01) at 200 µg/ml, 25.3 ± 2.2 % (*p*< 0.01) at 300 µg/ml, 18.3 ± 0.5 % (*p*< 0.01) at 400 µg/ml, and 3.4 ± 0.2 % (*p*< 0.01) at 500 µg/ml of ACE at 48 h (Fig. 2B) and by 67.3 ± 3.5 % (*p*< 0.01) at 100 µg/ml, 35.2 ± 1.1 % (*p*< 0.01) at 200 µg/ml, 23.2 ± 1.2 % (*p*< 0.01) at 300 µg/ml, 7.3 ± 0.3 % (*p*< 0.01) at 400 µg/ml, and 3.3 ± 0.1 % (*p*< 0.01) at 500 µg/ml of ACE at 72 h (Fig. 2C) in a dose-dependent manner. The survival of AGS cells was also evaluated using the CCK method. ACE treatment reduced the cellular viability by 84.4 ± 6.7 % at 100 µg/ml, 59.0 ± 3.3 % (*p*< 0.01) at 200 µg/ml, 38.8 ± 2.7 % (*p*< 0.01) at 300 µg/ml, 27.9 ± 2.9 % (*p*< 0.01) at 400 µg/ml, and 16.5 ± 1.4 % (*p*< 0.01) at 500 µg/ml (Fig. 2D). In addition, cell cycle analysis and mitochondrial membrane depolarization experiments were conducted to examine the induction of apoptosis by ACE. With ACE treatment, the proportion of sub G1 phase was increased and mitochondrial membrane was depolarized (Fig. 3). These results suggest that ACE inhibits the growth of AGS cells and that these effects are related to apoptosis induction.

### Relationship of ACE-induced apoptosis with mitochondrial pathway activation in AGS gastric cancer cells

**Figure 4:**
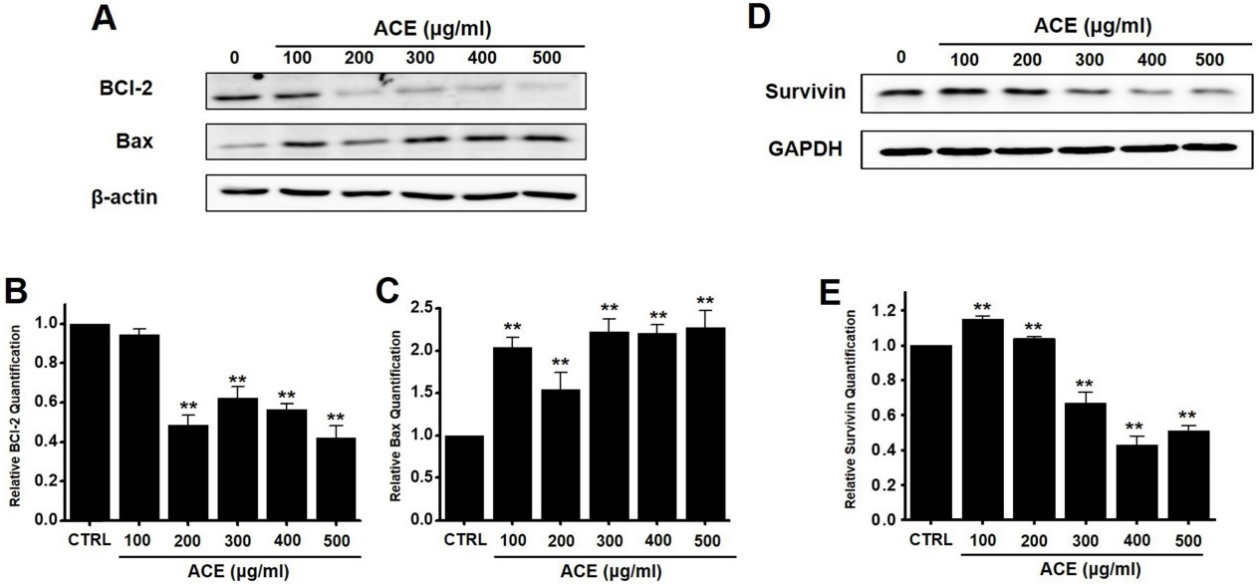
Effects of ACE on BCl-2, Bax, and survivin levels in AGS cells. (A) Western blot analysis results showing the downregulation and upregulation of Bcl-2 and Bax expression, respectively. (B) Bcl‑2 and (C) Bax expression normalized with respect to that of β‑actin. (D) Survivin expression is downregulated. (E) Survivin expression normalized with respect to that of GAPDH. Results are presented as the mean ± SEM. **p < 0.01. β-Actin and GAPDH are used as the loading controls. ACE, *Alisma canaliculatum* extract; CTRL, control.

We also analyzed whether the anti-apoptotic protein, Bcl-2, and pro-apoptotic protein, Bax, were involved in apoptosis following ACE treatment. Western blot analysis revealed that the levels of Bcl-2 decreased after treatment with ACE, whereas those of Bax increased (Fig. 4A-4C). In addition, the expression of survivin, an inhibitor of apoptosis, was decreased after ACE treatment (Fig. 4D and 4E). These results indicate that ACE-induced apoptosis was related to mitochondrial pathway activation in AGS cells.

### Relationship of ACE-induced apoptosis with caspase activation in AGS gastric cancer cells

**Figure 5:**
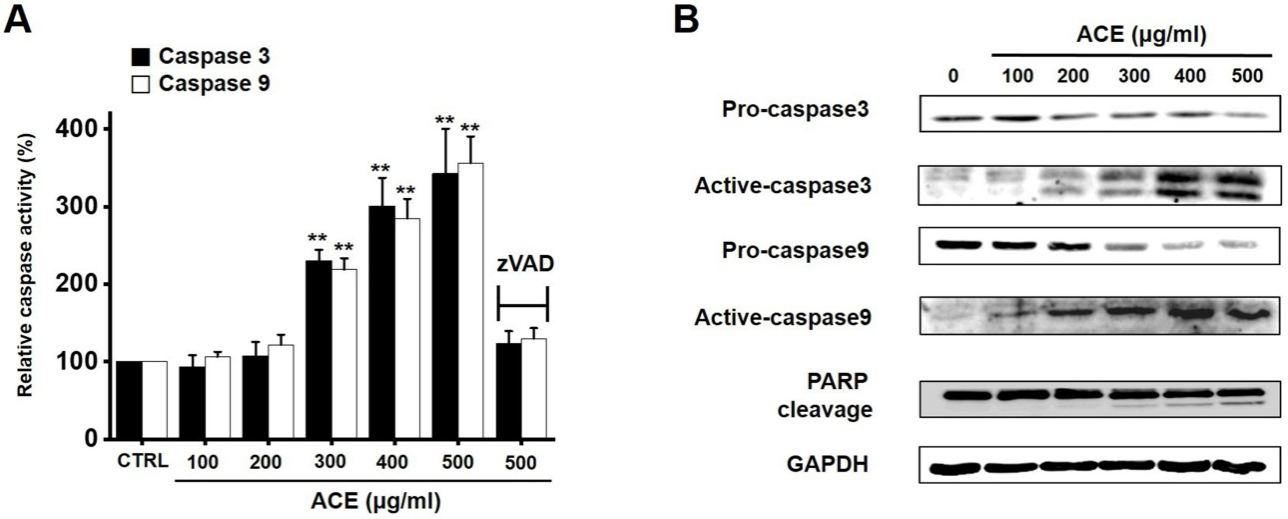
Involvement of caspase mechanism in ACE-treated AGS cells*.* (A) ACE dose-dependently increases the activities of caspase-3 and -9. (B) Western blotting results showing the involvement of caspase activation. Results are presented as the mean ± SEM. **p < 0.01. GAPDH is used as the loading control. ACE, *Alisma canaliculatum* extract; CTRL, control; zVAD, carbobenzoxy-valyl-alanyl-aspartyl-[O-methyl]- fluoromethylketone.

Caspases are important mediators of apoptosis. Moreover, the cleavage product of poly (ADP-ribose) polymerase (PARP) functions as a sign of apoptosis 16. ACE increased the caspase-3 and -9 activation dose-dependently, and zVAD-fmk inhibited this activation (Fig. 5A). In addition, western blot analysis suggested that ACE downregulated the expression levels of pro-caspase-3 and -9 and upregulated the levels of not only their active forms but also PARP cleavage (Fig. 5B). These results indicate that ACE-induced apoptosis was related to caspase activation in AGS cells.

### Regulation of the mitogen-activated protein kinase (MAPK) signaling pathway by ACE in AGS gastric cancer cells

**Figure 6:**
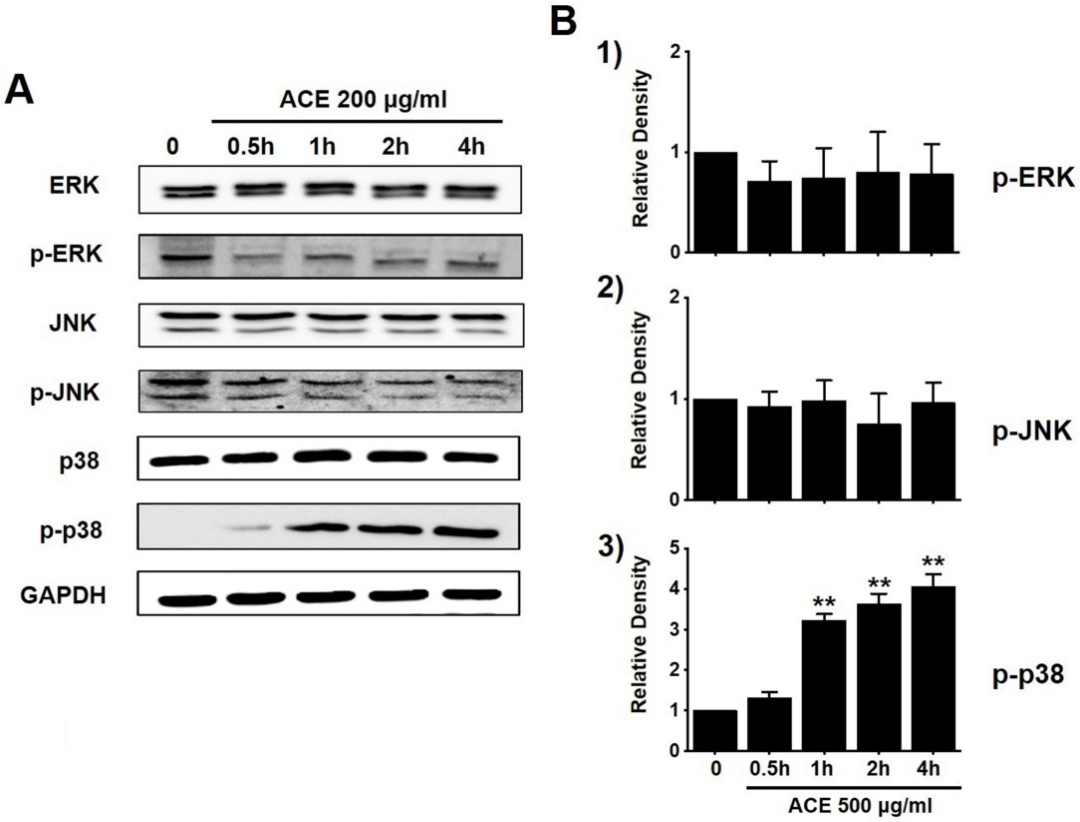
Involvement of ERK, JNK, and p38 proteins of the MAPK signaling pathway in ACE-treated AGS cells*.* (A) Western blotting results showing the phosphorylation of ERK, JNK, and p38. (B) Phosphorylated levels are indicated as band densities relative to those of GAPDH. Results are presented as the mean ± SEM. **p<0.01. ACE, *Alisma canaliculatum* extract; CTRL, control; ERK, extracellular signal regulated kinase; JNK, c‑Jun N‑terminal kinase.

To investigate the effects of ACE on the MAPK signaling pathway, we investigated the phosphorylation of MAPK proteins (extracellular signal regulated kinase (ERK), JNK, and p38) with western blotting. The phosphorylation of p38 increased with ACE treatment (200 μg/ml) (Fig. 6). These results indicate that ACE induced the apoptosis of AGS cells by modulating the MAPK signaling pathway.

### Regulation of intracellular ROS generation by ACE in AGS gastric cancer cells

**Figure 7:**
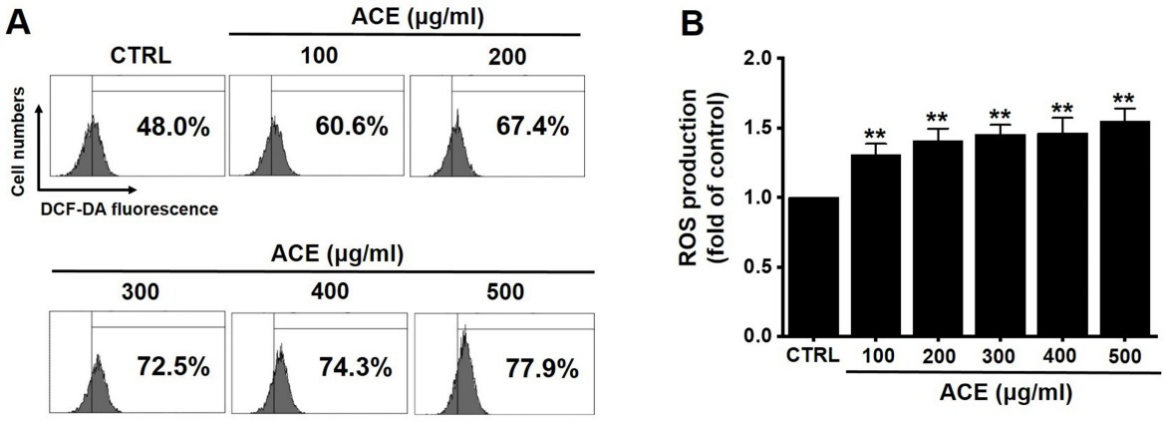
ACE increases ROS levels in AGS cells. (A) ROS levels measured using DCF-DA. (B) ROS levels are expressed as the percentage of ROS in untreated controls. Results are presented as the mean ± SEM. **p<0.001. ACE, *Alisma canaliculatum* extract; CTRL, control.

Since ROS also play a key role in apoptosis, we investigated whether ACE increased ROS levels in AGS cells. Flow cytometry results showed that ACE increased ROS levels (Fig. 7). These results indicate that ACE induced AGS cell apoptosis by ROS generation.

## Discussion

Traditional medicine is an ancient form of therapy that treats not only an illness or ailment but also the entire body holistically 17. *A. canaliculatum* is a plant used as a traditional medicine in Asia 5 and exerts many positive effects 6-10. It is also effective against cancer cells and can inhibit the metastasis of breast cancer 11. In addition, the present study shows that ACE induces apoptosis in AGS gastric cancer cells.

Apoptosis is mediated in eukaryotic cells through extrinsic and intrinsic pathways, and caspase cascade plays a key role in this process. The extrinsic pathway is initiated by the activation of caspase-8, which is triggered by the combination of death ligand to the death receptor present in the cell membrane, followed by the activation of caspase-3 and -7 18,19. In contrast, the intrinsic pathway, which begins around the mitochondria, forms an apoptosome and then promotes the activity of caspase-9 and caspase-3/-7 to cause apoptosis 20, 21. The inhibition of apoptosis is induced by terminating the activity of caspases, which is elicited by proteins belonging to the inhibitor of apoptosis protein family 22, 23. In the present study, ACE suppressed the proliferation of AGS cells (Fig. 2), induced the increase proportion of the sub-G1 phase, and depolarized the membrane potential of mitochondria (Fig. 3). ACE-induced cell death was associated with Bax increase and Bcl-2 decrease levels (Fig. 4); it also activated caspase-3 and -9 (Fig. 5). In addition, ACE reduced the expression levels of survivin (Fig. 4). Moreover, ACE-induced apoptosis was related to the MAPK signaling pathway (Fig. 6). ACE also increased the production of ROS (Fig. 7). These observations show that ACE induced apoptosis is dependent on caspase and mitochondrial pathways in AGS cells.

Ion channels play an important role in the formation and death of cancer. They are related with cancer cell growth, migration, and metastasis. Ion channels are also known to be critical components in apoptosis 24, 25. Among the various ion channels, TRPM7, TRPM2, and TRPC6, are known to be involved in AGS cell apoptosis 26-29. Thus, these ion channels may be targeted for gastric cancer treatment. TRPM7 is essential for the survival of AGS cells 26, 27, and TRPM2 has the ability to control the invasion of gastric cancer cells 28. In addition, the suppression of TRPC6 is known to inhibit gastric cancer formation 29. However, only a few studies have investigated the relevance of ion channels in the control mechanism of traditional medicine in gastric cancer apoptosis. Therefore, in the future, we should study the role of ion channel in gastric cancer apoptosis by ACE.

MAPKs are related with different cellular mechanisms including apoptosis 30. Therefore, they are known to be involved in cancer cell therapy mechanisms 31. In the present study, we found that ACE activated the p38 pathway, and it suggests that the modulation of MAPK cascades induces the apoptosis of AGS cells.

In conclusion, ACE inhibits the growth of AGS cells, induces the increase proportion of sub-G1 phase, and depolarizes the membrane potential of mitochondria. We found ACE-induced apoptosis to be associated with BCl-2 and survivin decreases and Bax increase levels; it also activated caspase-3 and -9 and MAPKs. Moreover, ACE increased the production of ROS. Thus, the present study on ACE may markedly contribute to the development of gastric cancer treatments.
